# From immobilized cells to motile cells on a bed-of-nails: effects of vertical nanowire array density on cell behaviour

**DOI:** 10.1038/srep18535

**Published:** 2015-12-22

**Authors:** Henrik Persson, Zhen Li, Jonas O. Tegenfeldt, Stina Oredsson, Christelle N. Prinz

**Affiliations:** 1 Division of Solid State Physics, Lund University, Box 118, 22100 Lund, Sweden; 2 NanoLund, Lund University, Box 118, 22100 Lund, Sweden; 3 Department of Biology, Lund University, Sölvegatan 35B, 223 62 Lund, Sweden; 4 Neuronano Research Center, Lund University, Sölvegatan 19, 221 84 Lund, Sweden

## Abstract

The field of vertical nanowire array-based applications in cell biology is growing rapidly and an increasing number of applications are being explored. These applications almost invariably rely on the physical properties of the nanowire arrays, creating a need for a better understanding of how their physical properties affect cell behaviour. Here, we investigate the effects of nanowire density on cell migration, division and morphology for murine fibroblasts. Our results show that few nanowires are sufficient to immobilize cells, while a high nanowire spatial density enables a ”bed-of-nails” regime, where cells reside on top of the nanowires and are fully motile. The presence of nanowires decreases the cell proliferation rate, even in the “bed-of-nails” regime. We show that the cell morphology strongly depends on the nanowire density. Cells cultured on low (0.1 μm^−2^) and medium (1 μm^−2^) density substrates exhibit an increased number of multi-nucleated cells and micronuclei. These were not observed in cells cultured on high nanowire density substrates (4 μm^−2^). The results offer important guidelines to minimize cell-function perturbations on nanowire arrays. Moreover, these findings offer the possibility to tune cell proliferation and migration independently by adjusting the nanowire density, which may have applications in drug testing.

During recent years, vertical nanowire arrays have received increasing attention for their possible use in life sciences^1^,^2^,^3^,^4^, as electrodes^5^,^6^,^7^, biosensors^8^,^9^,^10^,^11^,^12^,^13^,^14^,^15^, as well as for axonal guidance^16^,^17^, cell injections^18^,^19^,^20^,^21^,^22^ and anti-bacterial properties^23^,^24^,^25^. The rapidly expanding number of nanowire applications calls for a better understanding of the interactions between cells and nanowires, and, though steadily increasing, the number of papers studying cell-nanowire interactions remains low^4^. Some studies suggest that nanowires have little effect on cells, e.g. analyses of cellular mRNA content have shown no or limited changes in gene expression for cells cultured on nanowires compared to cells cultured on flat substrates^18^,^26^. Similarly, cell functions such as protein expression and enzymatic activity have been shown to be unaffected by the presence of vertical nanowires on the substrate^27^. The effects of nanowires on the cell membrane are not well understood either and seem to depend on cell type, nanowire density, interaction time span, and position of the nanowires with respect to the cell^28^,^29^,^30^,^31^. Nanowires have been shown to promote neuronal adhesion and axonal growth^1^,^17^,^32^,^33^,^34^, which has recently been attributed to an enhanced laminin adsorption on nanowires caused by curvature effects^35^. The presence of nanowires has also been shown to increase the number of cells in the S phase of the cell cycle and to up-regulate focal adhesion formation^36^. Systematic studies of how different aspects of nanowire geometry, such as density, length or diameter, are very valuable for developing and further improving nanowire-based applications. It has for instance been shown that nanowire spacing can be used to guide stem cell differentiation^37^ and tailoring nanowire length for optimal transfection was a key aspect in the work performed by Shalek *et al.*^38^ We have recently studied the effects of nanowire length on cell migration and division and found that cell motility and proliferation decreased with increasing nanowire length^29^. In that study, we showed that for an intermediate length of 4 μm, the nanowires have a limited effect on cells, making it possible to study the effects of other geometrical nanowire parameters on cell behaviour. Performing studies of how nanowire array geometry affects cell behaviour will improve our understanding of these interactions and better tailor future nanowire-based applications. Moreover, it may contribute to developing new applications of nanowires in cell biology.

Here, we used 4 μm long gallium phosphide (GaP) nanowire arrays and investigated the effects of nanowire density on cell behaviour, with emphasis on morphology, motility and proliferation. We used arrays of vertical GaP nanowires (80 nm in diameter) with a random spatial distribution (corresponding to widely used arrays in past studies^14^,^21^,^38^,^39^) and varied the average nanowire density from very low (0.1 μm^−2^) to high (4 μm^−2^). We cultured mouse fibroblasts (L929) on these substrates and used phase holographic time-lapse imaging^29^,^40^ to investigate cell motility and division. Fluorescence microscopy was used to study cell morphology and scanning electron microscopy (SEM) was used to investigate the cell-nanowire interface.

## Results

**Figure 1:**
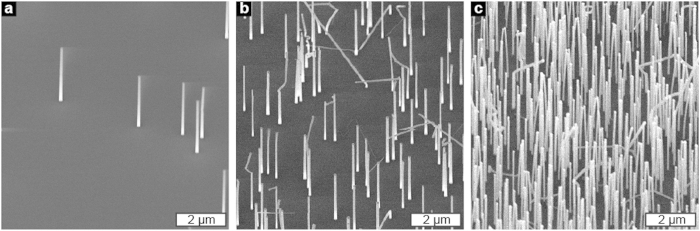
Scanning electron microscopy images of substrates with 0.1 nanowire μm^−2^ (**a**), 1 nanowire μm^−2^ (**b**) and 4 nanowires μm^−2^ (**c**). Tilt 30°.

Murine fibroblasts were cultured on substrates with vertical GaP nanowire arrays (Fig. 1) with a diameter of 80 nm and an average nanowire density ranging from 0.1 μm^−2^ (corresponding to an average distance of 3 μm between nanowires) to 4 μm^−2^ (corresponding to an average distance of 0.5 μm between nanowires).

### Migration

**Figure 2:**
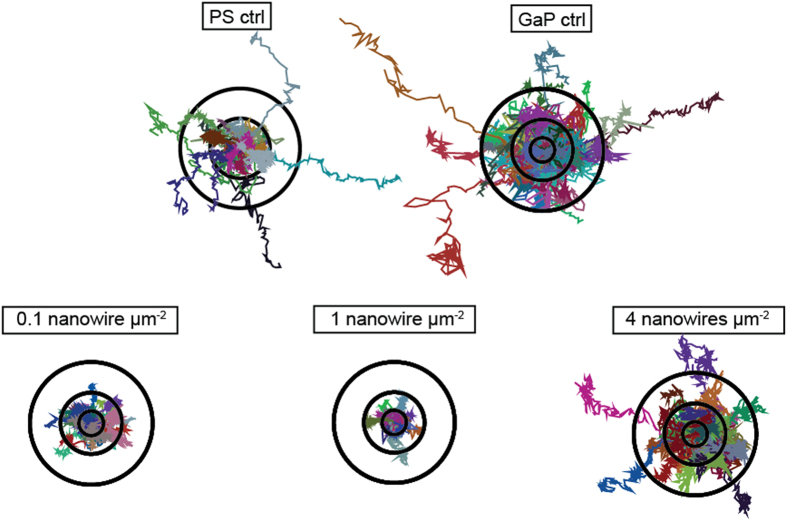
Path of L929 cells migrating for 20 h on the different substrates; Polystyrene flat control substrates (PS ctrl), GaP flat control substrates (GaP ctrl) and nanowire substrates of various densities. Black circles have radii of 10, 25 and 50 μm, respectively. Cell diameter is approximately 25 μm.

**Figure 3:**
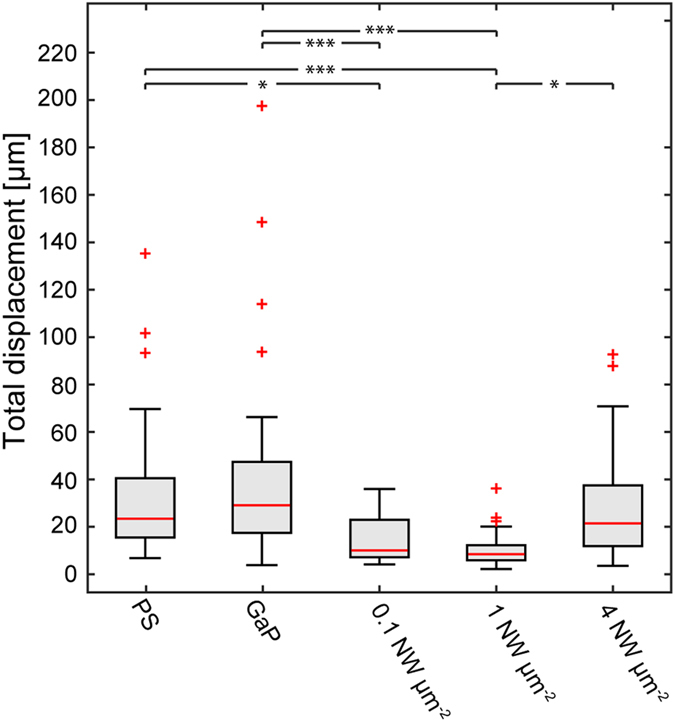
Total displacement of cells traced over 20 h on the different substrates. The box displays the 25^th^ and 75^th^ percentile while the bars denote the highest and lowest value. Horizontal, red lines denote sample medians and red crosses show outliers, defined as data points outside 2.7 standard deviations (99.3% of the data). ***p < 0.001; *p < 0.05 (multiway analysis of variance). NW: nanowires.

Using phase holographic microscopy, we captured 24–72 h long time-lapse movies of cells on the substrates (see Supplementary information, Movie S1-S5). For each individual cell, the centre of mass was determined and followed over time. The resulting track traces of the cells on the different substrates are shown in Fig. 2, visualizing the cell migration during the first 20 h after seeding. The corresponding total displacement is summarized in Fig. 3. Only the first 20 h were chosen in order not to bias the data toward slower moving cells, as faster cells can leave the field of view. The cell motility is lower on all nanowire substrates compared to Polystyrene (PS) and GaP controls except on 4 μm^−2^ substrates, where cells move to a similar degree as those on PS and GaP (Figs 2 and 3).

Cells on the lower-density substrates (0.1 μm^−2^ and 1 μm^−2^) are completely immobile. In contrast, cells on 4 nanowires μm^−2^ arrays move in a similar manner compared to cells on flat control substrates, both with regards to total distance and migration pattern, suggesting a ”bed-of-nails” regime, where the nanowires are dense enough to support the cells and are perceived as a continuous substrate.

**Figure 4:**
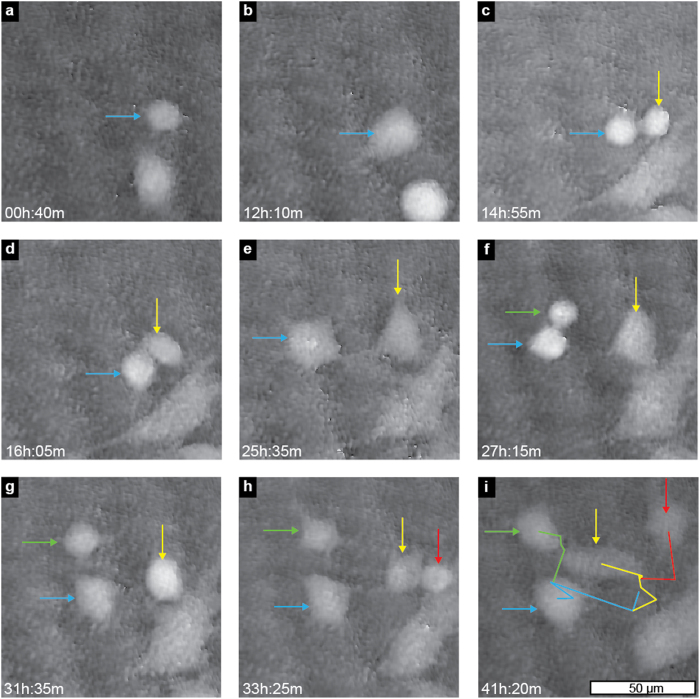
Selected frames from Movie S6 (Supplementary information), showing the mode of migration of cells on 0.1 nanowire μm^−2^ substrates. The cells remain stationary for extended periods of time until a division event releases one or both daughter cells from the nanowires. In the images, the cell labelled with a blue arrow (**a**,**b**) divides (**c**) and the two daughter cells (blue and yellow arrows) migrate over a short distance (**d**,**e**). When the cell labelled with a blue arrow divides (**f**), one of the daughter cells (green arrow) migrates whereas the other daughter cell (blue arrow) remains stationary. Four hours later, the cell labeled with a yellow arrow divides (g,h) and the resulting daughter cells migrate a short distance in the last 8 h of the experiment. In the final frame (**i**), the movement of the cells during the entire time-lapse is shown as coloured lines. Note that these particular cells move on the order of 20 μm over more than 40 h. Time stamps refer to time after seeding.

On 0.1 μm^−2^ density substrates, the cells are able to move for a short distance after division, until they encounter nanowires that inhibit their migration (see Fig. 4 and Supplementary information, Movie S6).

### Proliferation

**Figure 5:**
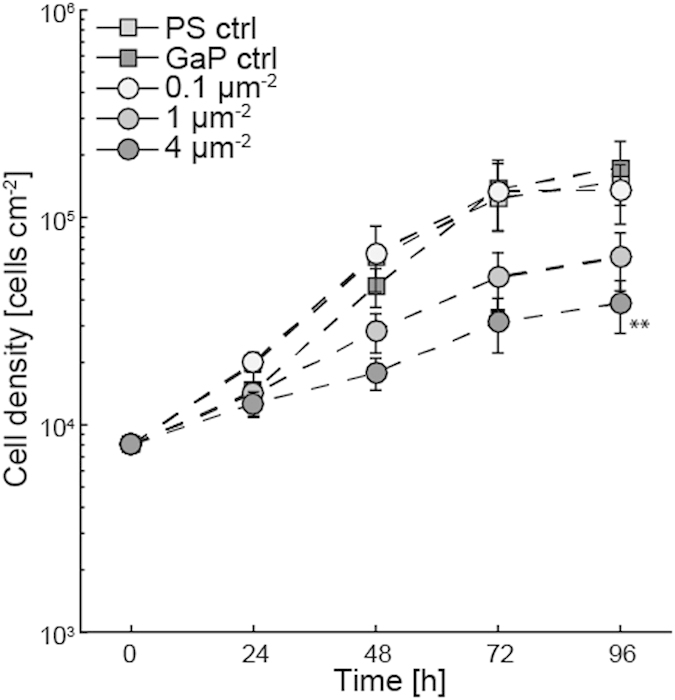
Cell number (mean ± S.E.M., n = 3) on the different nanowire density substrates, determined from phase holographic microscopy imaging, plotted as a function of time after seeding. **denotes a statistically significant difference with p < 0.01 compared to PS control as determined using Student’s t-test with Bonferroni correction for multiple comparisons.

The cell density on the different substrates was determined every 24 h for a period of 96 h to generate growth curves (Fig. 5). The rate of cell proliferation is lower on nanowire substrates with medium (1 μm^−2^) and high density (4 μm^−2^) while the proliferation rate of cells cultured on the low-density samples (0.1 μm^−2^) is comparable to both flat controls (PS and GaP).

### Morphology

**Figure 6:**
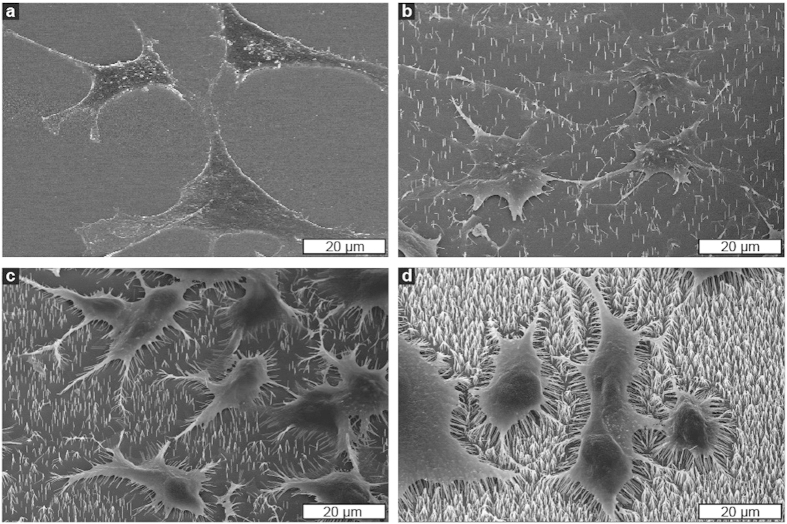
Scanning electron microscopy images (tilt 30°) showing the morphology of L929 cells cultured on plain GaP (**a**), on nanowires with a density of 0.1 μm^−2^ (**b**), 1 μm^−2^ (**c**) and 4 μm^−2^ (**d**). Cells on a substrate with 0.1 nanowire μm^−2^ adhere to the substrate between nanowires, with the nanowires protruding into the cells. Cells cultured on a high nanowire density (4 μm^−2^) lie on top of the nanowires.

**Figure 7:**
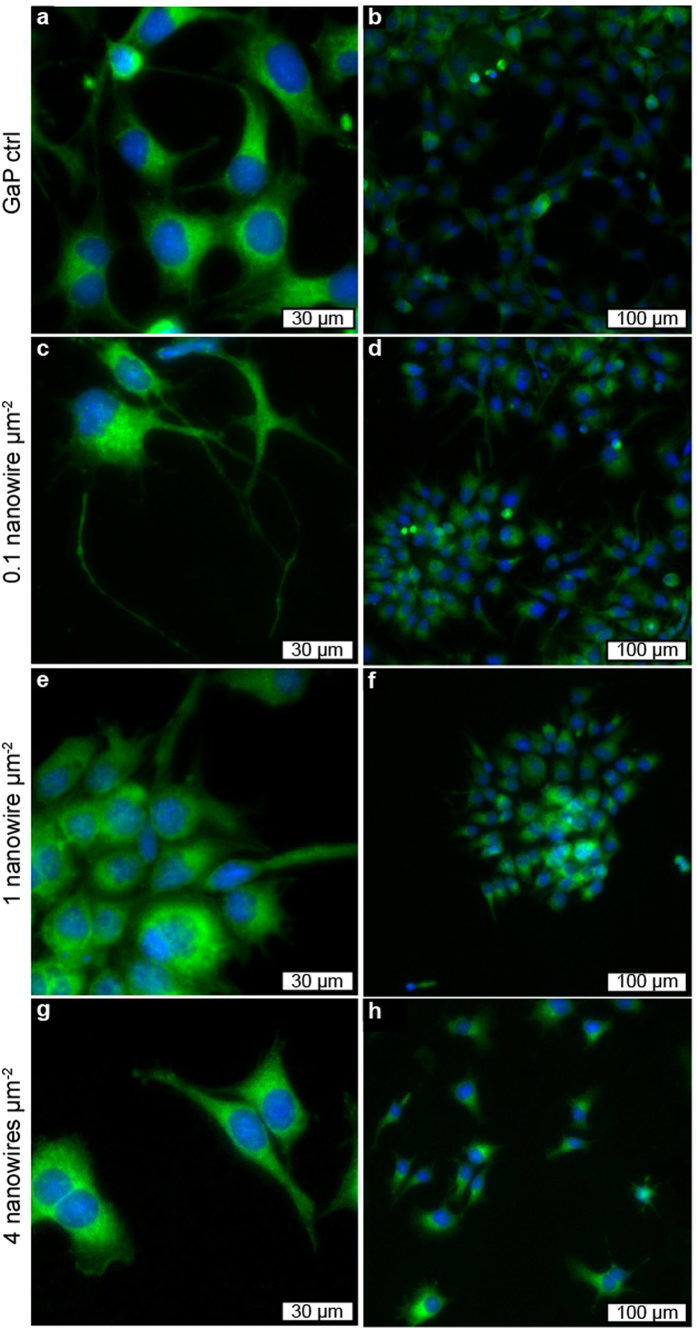
Fluorescence microscopy images showing cell morphology and distribution after 96 h culture on nanowire substrates with different densities. In the fluorescence images, actin is labelled with FITC-conjugated phalloidin (green) and DNA is labelled with Hoechst 33342 (blue).

SEM (Fig. 6) was used to study the cell-nanowire interface and fluorescence microscopy was used to assess the morphology of cells on the substrates (Fig. 7). Cells adopt an irregular, elongated morphology with thin protrusions on the low-density substrates (0.1 μm^−2^) and to some extent on medium-density substrates (1 μm^−2^) in contrast to the polygonal morphology on the flat control substrates (PS and GaP). The time-lapse movies revealed that the cells extend longer and longer protrusions (Supplementary Movie S7).

On the low and medium nanowire density substrates (0.1 and 1 μm^−2^), cells form isolated cell colonies caused by a lack of migration preventing cells from spreading after division (Fig. 7d,f). In these clusters, most fibroblasts at the edges display long and thin protrusions (Figs 6b,c and 7c,e). The SEM images of cells on the same low-density substrate (see for instance Fig. 6b) show that few (50–100), nanowires are required to immobilize the cells on the substrate.

Cells on 4 μm^−2^ nanowire arrays are morphologically similar to cells on control substrates (Fig. 7g,h), and the SEM images confirm the “bed-of-nails” regime, with cells lying on top of the nanowires (Fig. 6d).

**Figure 8:**
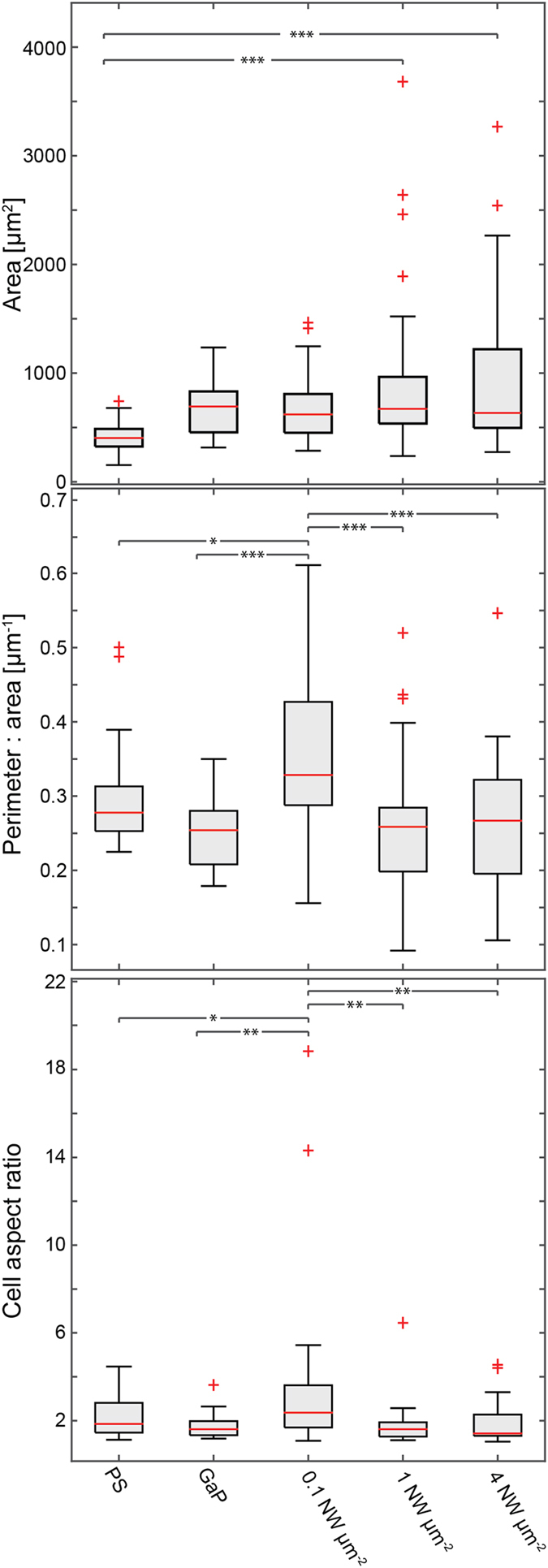
Analysis of key aspects of cell morphology on the different substrates (n = 3). The cell aspect ratio is calculated as the ratio between major and minor axis of ellipses fitted to each cell. The data show that cells on the substrates with 1 and 4 nanowires μm^−2^ are significantly larger than cells on the PS control and that cells cultured on the substrate with 0.1 nanowires μm^−2^ are significantly more elongated and have a higher perimeter-to-area ratio compared to cells on all other substrates. Asterisks denote statistically significant differences as determined using multivariate analysis of variance (ANOVA). ***p < 0.001, **p < 0.01, *p < 0.05.

We have quantified the cell area, perimeter-to-area ratio and elongation (aspect ratio, defined as the ratio between major and minor axis of a fitted ellipse) on the different substrates (Fig. 8). Cells on 1 and 4 nanowires μm^−2^ have a similar elongation and perimeter-to-area ratio compared to cells on control substrates. Our results also show that cells on substrates with 0.1 nanowires μm^−2^ are more elongated and present an increased perimeter-to-area ratio, which is reflected by the long and thin cell protrusions that these cells often exhibit. Representative cells from the different substrates are shown in Supplementary Figure S2.

### Nuclear morphology

**Figure 9:**
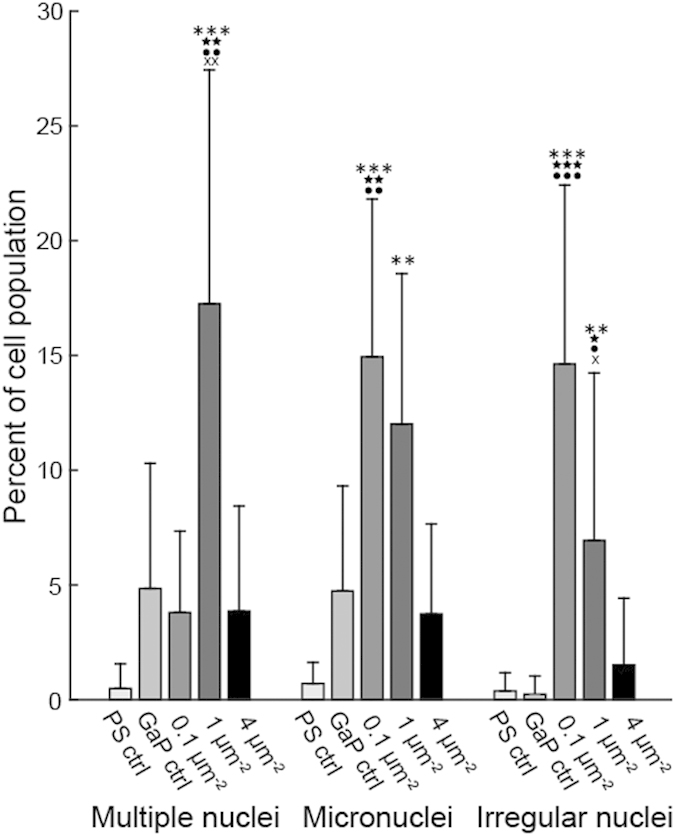
Occurrence of nuclear abnormalities on nanowire substrates after 96 h of culture. The terms multiple nuclei, micronuclei and irregular nuclei are defined in the *Methods* section. Mean ± S.E.M., n = 3, at least 100 cells per sample were analysed. Symbols above bars denote statistically significant differences as determined using multivariate analysis of variance (ANOVA). *denotes difference compared to PS, ★ denotes difference compared to GaP, ● denotes difference compared to high nanowire density (4 μm^−2^) and X denotes difference compared to low nanowire density (0.1 μm^−2^). Three symbols correspond to p < 0.001, two symbols to p < 0.01 and one symbol to p < 0.05.

We have previously reported that cell populations cultured on 1 μm^−2^ dense nanowire arrays have a higher proportion of cells with abnormal nuclei, especially when cultured on long nanowires^29^. In order to investigate the dependence of nuclear abnormalities on nanowire density, we determined the number of cells with multiple cell nuclei, cell nuclei with irregular morphology, as well as the number of micronuclei per cell (Fig. 9) (see methods for definitions). Both multinuclear cells and micronuclei are more prominent on flat GaP and all nanowire substrates compared to PS control substrates and a significantly higher number of multinuclear cells can be seen for cells on the medium-density samples. Cells with irregular nuclei and micronuclei are more common on low and medium-density samples.

## Discussion

**Figure 10:**
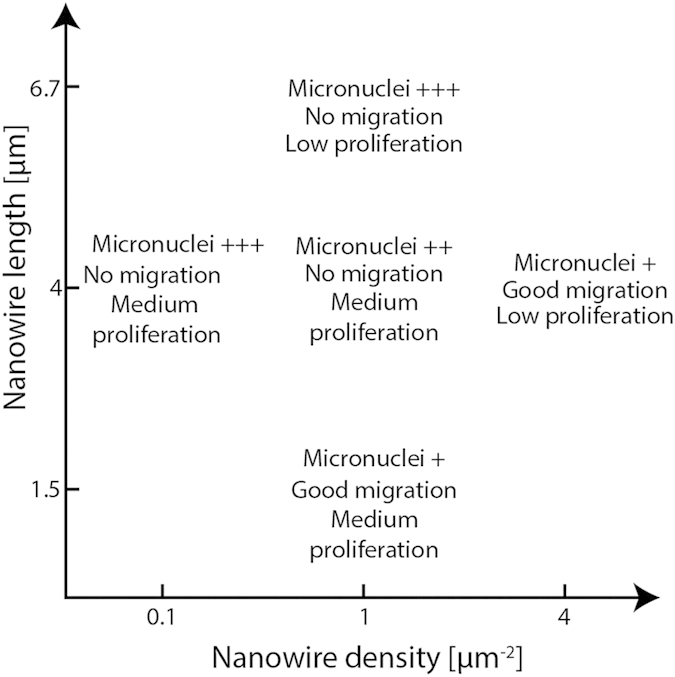
Effect of nanowire length and density on cell proliferation, migration and micronuclei formation, compiled from the present paper and our previous work^29^.

We have studied murine fibroblasts cultured on GaP nanowire substrates with varying density, from 0.1 to 4 nanowires μm^−2^. Time lapse images indicated that the cells remained viable for the duration of these experiments, with continued proliferation for at least 96 h and migration observed up to 72 h. This study of key aspects of cell behaviour on nanowires with different physical parameters is a continuation of our previous work where we varied nanowire length^29^ instead of density. To facilitate comparisons to our previous findings, the key findings of the current and previous work have been summarized in Fig. 10.

Our current results show that the cell mobility is decreased on 0.1 and 1 μm^−2^ density nanowire arrays, which is in agreement with previous studies reporting the immobilization of cells using nanowires^29^,^41^. In contrast, on the 4 μm^−2^ density array, cells are motile to the same extent as cells on flat control substrates, suggesting that high-density nanowire arrays are perceived as flat substrates by the cells. Whether a specific cell type lies on top of nanowires of a given density or adheres to the substrate between them, is suggested to depend on the mechanical properties of the cell, such as membrane stiffness and cytoskeletal rigidity^30^,^42^. In our case, the minimum nanowire density necessary for reaching the “bed-of-nails” regime is somewhere between 1 and 4 nanowires μm^−2^ for L929 fibroblasts, which is in line with previous findings showing cells lying on top of nanowires at densities above 0.3 μm^−2^ for HEK293^36^ and C3H10T1/2 cells^37^, and 1 μm^−2^ for primary neurons^8^.

Our results on cell proliferation on 1 μm^−2^ substrates agree well with our previously published results^29^. The cell proliferation rate on 4 μm ^2^ nanowire arrays is significantly lower than the one on flat controls, indicating that the high-density substrates cannot be considered equivalent to flat substrates in general although they have no effect on cell motility. The lower proliferation rate observed on these substrates could possibly be attributed to an increased cell adhesion, as it has been reported that cell adhesion is stronger on nanowires and pillars compared to flat substrates^36^,^43^,^44^,^45^ and that there is an increased adsorption of the adhesion promoting protein laminin on nanowires^35^. However, a stronger cell attachment on the dense nanowire array seems in contradiction with the high cell motility observed on this substrate. Further studies, such as using migration or mitosis blocking compounds, would help elucidate the mechanisms behind the observed effects on cell division. Different substrates with high aspect ratio nanostructures have been shown to support cell proliferation, in some cases at unchanged rates^46^, but often at decreased rates^18^,^24^,^29^.

We show that when cells are completely immobile on the substrate and are able to divide (1 μm^−2^ arrays), they are arranged in clusters on the substrate. Since cells are immobile and the seeding density was low, each cluster consists of daughter cells arising from a single cell (Fig. 7f). This could be of interest for testing the effects of drugs or particles on a single cell progeny. The formation of such clusters makes it possible to follow several generations originating in a single cell, as demonstrated in Supplementary information Movie S8.

Our results also show that cells on substrates with 0.1 nanowires μm^−2^ are very elongated, which is reflected by the long and thin cell protrusions that these cell often exhibit. Bonde *et al.* have previously reported an elongated cell morphology on low-density nanowire arrays^36^, although not to the same extent as in the present study. That study showed a 2% increase in cell aspect ratio for cells cultured on 0.13 nanowires μm^−2^ substrates compared to flat semiconductor control (no increase in cell aspect ratio when compared to glass), whereas our results indicate an increase of 55% for cells cultured on 0.1 nanowire μm^−2^ substrates compared to PS and an increase of 98% compared to cells on flat GaP. Long and narrow protrusions, similar to the ones found here, have previously been reported for mesenchymal stem cells cultured on dense nanowire arrays where cells reside on top of the nanowires^37^ as well as sparse nanowire arrays where cells are located on the substrate^47^. In those studies, the narrow protrusions were interpreted as stem cells differentiating into neurons. Kim *et al.* also reported similar long protrusions for primary endothelial cells (HUVEC) immobilised on sparse nanowire arrays^47^. They interpret the protrusions as an indication of the cells exploring the surface even though the cell body is immobilised by the nanowires. Similarly, Diu *et al.* observed tear-drop shaped morphologies, on high aspect ratio TiO_2_ nanowires^24^. They too attributed these elongated morphologies to migration attempts of partially immobilized cells. In our case, the cell body is pinned to the substrate and long protrusions are actively formed (see Supplementary Movie S7) (as opposed to cell remnants being left behind a migrating cell). We can therefore speculate that these long processes correspond to a cellular response to molecular cues, which normally influence fibroblast movement, which is hindered by the nanowires in the present case. SEM images further reveal the interactions between the cells and nanowires. Cells on the 0.1 μm^−2^ samples are in contact with the substrate and since the cells are thinner than the nanowire length, the nanowires protrude through the cells (Supplementary Figure S1). In agreement with the literature^28^, these protruding nanowires appear engulfed by cell membrane. Whether or not the membrane is penetrated or intact is an open question and several papers are addressing this question, both through experimental observations^28^,^48^,^49^,^50^ and through theoretical modelling^30^,^42^,^51^.

Our results show that multinuclear cells and micronuclei are more prominent on flat GaP and all nanowire substrates compared to PS control substrates with a distinct increase in multinuclear cells on the medium-density samples and a higher proportion of cells with irregular nuclei and micronuclei on low and medium-density samples. The formation of micronuclei and faulty nuclear divisions are biomarkers of genotoxic events that cause DNA-strand breaks^52^. The generation of micronuclei has previously been connected to reactive oxygen species (ROS), which induces formation of micronuclei even without cell division taking place^53^. In the present case, we can hypothesize that the formation of micronuclei is related to the increased production of ROS that we have previously observed in L929 cells grown on nanowire substrates as well as on plain GaP^29^. Another possible explanation for the occurrence of cells with multiple nuclei may be that, on nanowires, the cell cytoskeleton cannot readily perform the rearrangements required to achieve cytokinesis. The irregular appearance of some nuclei might stem from the nuclei being physically prevented from adopting their regular morphology due to the presence of nanowires. Another mechanism through which micronuclei can form is erroneous chromosome separation during cell division. Indeed, we found a correlation between the occurrence of micronuclei and the rate of cell proliferation on the different substrates. For instance, cells on the 0.1 μm^−2^ substrate proliferate at a rate four times higher than cells on the 4 μm^−2^ substrate and micronuclei are four times more frequent on the 0.1 μm^−2^ substrate. This also holds for the 1 μm^−2^ substrate, within the experimental margin of error. This may suggest that the rate of micronuclei formation depends on the cell proliferation rate rather than on the nanowire density *per se*.

In summary, we have investigated the effects of nanowire density on L929 mouse fibroblast behaviour and morphology. On low-density arrays, the cells are attached to the substrate and migration is non-existent. The lack of migration results in the presence of localized cell clusters where cells at the edges adopt morphologies with long, thin protrusions. On high-density arrays (4 nanowires μm^−2^), the cells are lying on top of the nanowires, in a ”bed-of-nails” regime and adopt a morphology that is similar to the one of cells on flat control substrates. Cells on this substrate are mobile to a degree comparable to cells on control substrates. The rate of cell proliferation decreases with increasing nanowire density and is significantly lower on 4 nanowires μm^−2^ compared to PS control. The present results open up for tuning cell motility and proliferation independently using arrays of nanowires. For instance, using 1 nanowire μm^−2^ arrays would result in moderately dividing but immobilized cells, producing scattered clusters of clonogenic cells, which can be useful in drug testing. On the other hand, using 4 μm^−2^ nanowire arrays would inhibit division but not migration, enabling tracking of individual cells for a long period of time in situations where the occurrence of cell division would disturb the measurements.

## Methods

### Nanowire arrays

GaP nanowires were grown using metal organic vapour phase epitaxy (Aix 200/4, Aixtron, Herzogenrath, Germany) from spark discharge-generated aerosol gold nanoparticles^54^ (80 nm diameter) deposited on double side polished GaP (111) wafers (Girmet Ltd, Moscow, Russia). The nanowires were grown from the gas precursors trimethyl-gallium and phosphine, following a protocol published earlier^55^, except for using a temperature of 550 °C for the annealing step instead of 470 °C. By controlling the total growth time, we could control the length of the nanowires, which was set to 3.9 ± 0.3 μm (mean ± standard deviation) for each sample. Because the growth rate of the nanowires is highly dependent on the density of seed particles, each substrate with a different particle density was grown separately after calibrating the density dependent growth rate. Arrays with an average nanowire surface density of 0.1 ± 0.02 μm^−2^, 1 ± 0.04 μm^−2^ and 4 ± 0.3 μm^−2^ were fabricated. The average size of the samples was 0.3 cm^2^.

### Cell culture

L929 mouse fibroblasts (DSMZ, Braunschweig, Germany) were cultured in polystyrene (PS) culture flasks in RPMI1640 medium supplemented with 10% fetal calf serum, 100 U/mL penicillin and 100 μg/mL streptomycin. Cell cultures were kept in a 37 °C incubator with 5% CO_2_ in humidified air. Before seeding cells on nanowire substrates, the cells were detached using trypsin and counted in a haemocytometer. For experiments involving phase holographic imaging (i.e. time-lapse and growth curves), we used substrates polished on both sides to enable imaging, as previously described^29^. Prior to cell culturing, the substrates were sterilized overnight using UV irradiation.

### Time-lapse imaging and cell tracking

The nanowire substrates were placed in 25 cm^2^ PS tissue culture flasks (one sample per flask) and the cells were suspended in warm medium and then added to the flasks in which the samples had been placed. Cells were seeded at a density of 15000 cm^−2^. Tissue culture plastic (PS) and plain GaP were used as control substrates. The flasks were filled with air containing 5% CO_2_ and sealed in order to maintain the pH of the medium. The flasks were then directly transferred to a phase holographic microscope (HoloMonitor M3, PHI AB, Lund, Sweden) kept in a 37 °C dry incubator where they were continuously imaged. Images were saved every 5 min for 24 or 72 h. For the samples with 4 nanowires μm^−2^, images were instead saved every 2 min to compensate for the fact that several images taken at this density had to be discarded due to high noise levels caused by light scattering from the nanowires, making holographic image reconstruction impossible. Note that the samples were all illuminated at the same frequency (governed by software), i.e. there is no difference in light exposure between time lapse images acquired using different intervals between image acquisition. For migration analysis, n = 3 (PS, 0.1 and 4 nanowires μm_–2_) or n = 4 (GaP and 1 nanowire μm_–2_) was used. In each case, at least 8 cells were traced unless a lower number of cells were present in the field of view (6–21 cells were traced per sample, with a mean value of 11 cells). For each sample type, at least 29 cells were analysed.

Phase holographic imaging is based on an interference pattern created by a long wavelength laser (633 nm)^56^ passing through the specimen. The image of the sample is restored from the interference pattern. Using a long-wavelength laser and double-side polished substrates makes it possible to investigate living cells on GaP nanowire substrates in a label-free manner^29^. The low intensity combined with the long wavelength of the laser light makes it possible to image cells for long periods of time with negligable photocytotoxicity. The method yields 3D images with high contrast, which allows for cell identification and tracking on the sample using the phase holographic microscope software (HStudio 2.6, PHI AB, Lund, Sweden)^40^,^57^. In short, cells are initially identified using local maxima above a user-defined intensity threshold (H-maxima, see e.g. ref. 58). These maxima are used as seeds and a watershed algorithm is used to identify the entire cell and calculate the centroid. Automated cell identification, combined with manual verification, was used to continuously track cells throughout the first 20 h of each movie. In order to ensure that the cell-cell contact events are negligible, we have used low cell densities and we have limited the analysis to 20 h. After 20 h, several cells started to leave the field of view and hence 20 h was chosen as the end point for analysis of the time lapse movies. Limiting tracking to 20 h ensures that differences in cell proliferation do not impact the migration analysis. To correct for possible sample drift, the average displacement across all cells for each time point was subtracted from the location of each individual cell. Migration was characterised by total displacement for cells (start point to end point) and analysed using multiway analysis of variance (ANOVA) as implemented by MatLab’s function *anovan( )* in the Statistics Toolbox (MatLab 8.4, Mathworks, Natick, USA).

### Growth curves

To monitor the proliferation of the cells during 96 h, cells were seeded at a density of 8000 cm^−2^ on double side polished nanowire substrates (n = 3) placed in 25 cm^2^ culture flasks (one sample per flask), and cultured in an incubator at 37 °C with 5% CO_2_ in humidified air. Flasks without a sample were used as controls. Once seeded, the cells were allowed to adhere for 3 h before the flasks containing samples were moved to the M3 phase holographic microscope (placed in a 37 °C dry incubator). After imaging, the flasks were returned to the standard incubator. This was repeated every 24 h up to 96 h after seeding. The cell number was determined using the microscope software (HStudio 2.6, PHI AB, Lund, Sweden). The automatic cell identification by the software was manually verified to ensure that the cell density was correctly measured. For quantification, cells in at least 3 fields of view were identified. For substrates with low cell density, more images were acquired. The least number of cells for one single time point was 84, at t = 0 h, but more commonly at least 200 cells and up to several thousand were identified for each sample and time point. Statistical analysis of the endpoint density was performed using Student’s t-test, comparing all samples to PS controls using Bonferroni correction.

### Fluorescence microscopy

After 96 h of culture, the growth curve samples were fixed for 20 min in 3.7% formaldehyde and rinsed in phosphate-buffered saline (PBS). Samples were incubated in PBS containing 1% (v/v) Tween20 and 1% bovine serum albumin (BSA) (w/v) (Sigma-Aldrich, St. Louis, USA) for 30 min. Cells were then labelled with fluorescein isothiocyanate (FITC)-conjugated phalloidin (Sigma-Aldrich, St. Louis, USA) at a concentration of 1.7 μg/μL in PBS containing 1% (v/v) Tween20 and 1% BSA (w/v) for 90 min. After washing with PBS, the samples were counter-stained using Hoechst 33342 (Invitrogen, Carlsbad, USA) at a concentration of 1 μg/mL in PBS for 1 min. After a final washing step in PBS, the samples were imaged in a standard fluorescence microscope (TE2000, Nikon, Tokyo, Japan) with standard wide field objectives and an iXon897 EMCCD camera (Andor, Belfast, UK). Cell morphology was evaluated by manually tracing the outline of at least 10 cells per sample (n = 3) in ImageJ (ImageJ 1.47v, National Institutes of Health, USA). Images were further analysed for deviating nuclear morphologies and micronuclei via manual counting using the Cell Counter plugin in ImageJ. Cell nuclei were divided into three categories: normal, multinuclear and irregular nuclei. Multinuclear cells were defined as cells containing either multiple nuclei or nuclei with several lobes, which appear as unseparated multiple nuclei. Irregular nuclei was defined as nuclei with sharp folds. Micronuclei were defined as miniscule satellite nuclei besides the main nucleus. Examples of these nuclear morphology categories are shown in Figures S3-S5. The fraction of cells containing micronuclei, multiple nuclei and/or irregular nuclei was quantified for n = 3 independent experiments. For each experiment, at least 100 cells were analysed. Both with regards to cell morphology and nuclear morphology, statistical analysis was carried out using ANOVA as implemented by MatLab’s function *anovan( )* (MatLab 8.4, Mathworks, Natick, USA).

### Scanning electron microscopy

After 96 h of culture, cells were fixed for 20 min in 3.7% formaldehyde, washed in PBS and then dehydrated in graded ethanol series (20%, 50%, 70%, 95%, 99% and absolute ethanol). Following air drying, the samples were sputter-coated with platinum (Polaron E5100 DC, Quorum Technologies, Laughton, UK) and imaged using a thermal field emission SEM (LEO1560, Carl Zeiss SMT GmbH, Oberkochen, Germany).

## Additional Information

**How to cite this article**: Persson, H. *et al.* From immobilized cells to motile cells on a bed-of-nails: effects of vertical nanowire array density on cell behaviour. *Sci. Rep.* **5**, 18535; doi: 10.1038/srep18535 (2015).

## Supplementary Material

Supplementary Information

Supplementary Movie 1

Supplementary Movie 2

Supplementary Movie 3

Supplementary Movie 4

Supplementary Movie 5

Supplementary Movie 6

Supplementary Movie 7
