# Supplementary material for: From immobilized cells to motile cells on a bed-of-nails: effects of vertical nanowire array density on cell behaviour
Source: Sci Rep. 2015 Dec 22;5:18535. doi: 10.1038/srep18535 (PMC4686997; doi:10.1038/srep18535)
Supplement: Supplementary Information [file srep18535-s1.doc]

From immobilized cells to motile cells on a bed of nails: effects of vertical nanowire array density on cell behaviour

Henrik Persson^1,2,#^, Zhen Li^1,2^, Jonas O. Tegenfeldt^1,2^, Stina Oredsson^2,3^, and Christelle N. Prinz^1,2,4 *^

^1^ Division of Solid State Physics, Lund University, Box 118, 22100 Lund, Sweden

^2^ NanoLund, Lund University, Box 118, 22100 Lund, Sweden

^3^ Department of Biology, Lund University, Sölvegatan 35A, 223 62 Lund, Sweden

^4^ Neuronano Research Center, Lund University, Sölvegatan 19, 221 84 Lund, Sweden

^#^ present address: Experimental Medical Science, Lund University, Box 118, 22100 Lund, Sweden


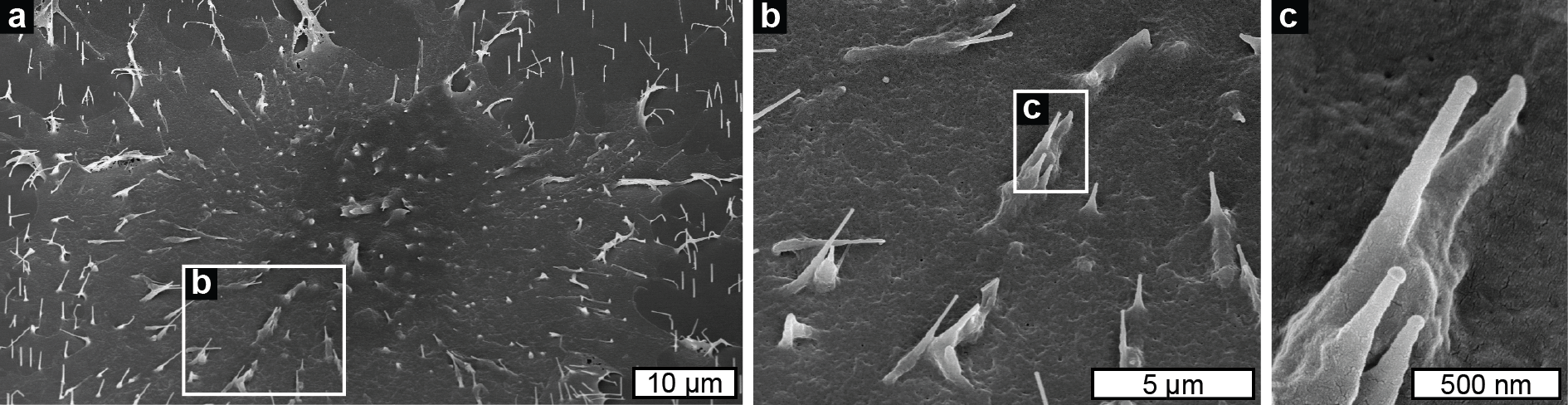


Figure S1 SEM image of fibroblast cultured for 96 h on a substrate with 0.1 nanowire µm^‑2^. The nanowires extend through the back of the cells and are covered with membrane. Tilt 30°.


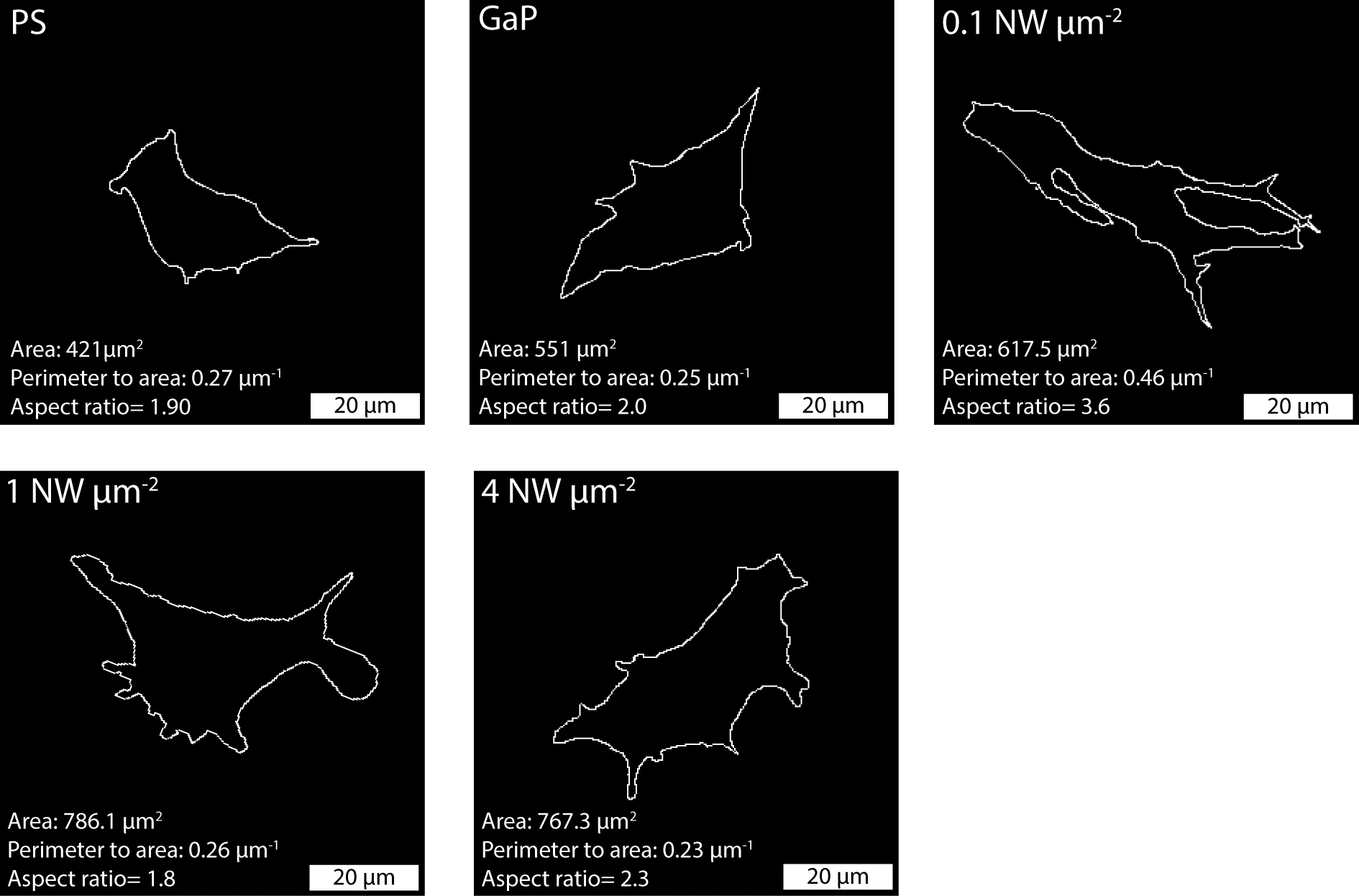


Figure S2 Representative cell morphologies for L929 cells cultured on the different substrates, as labelled in the figure. Cells were selected with area, perimeter-to-area ratio and aspect ratio values close to the sample mean as presented in Figure 8.


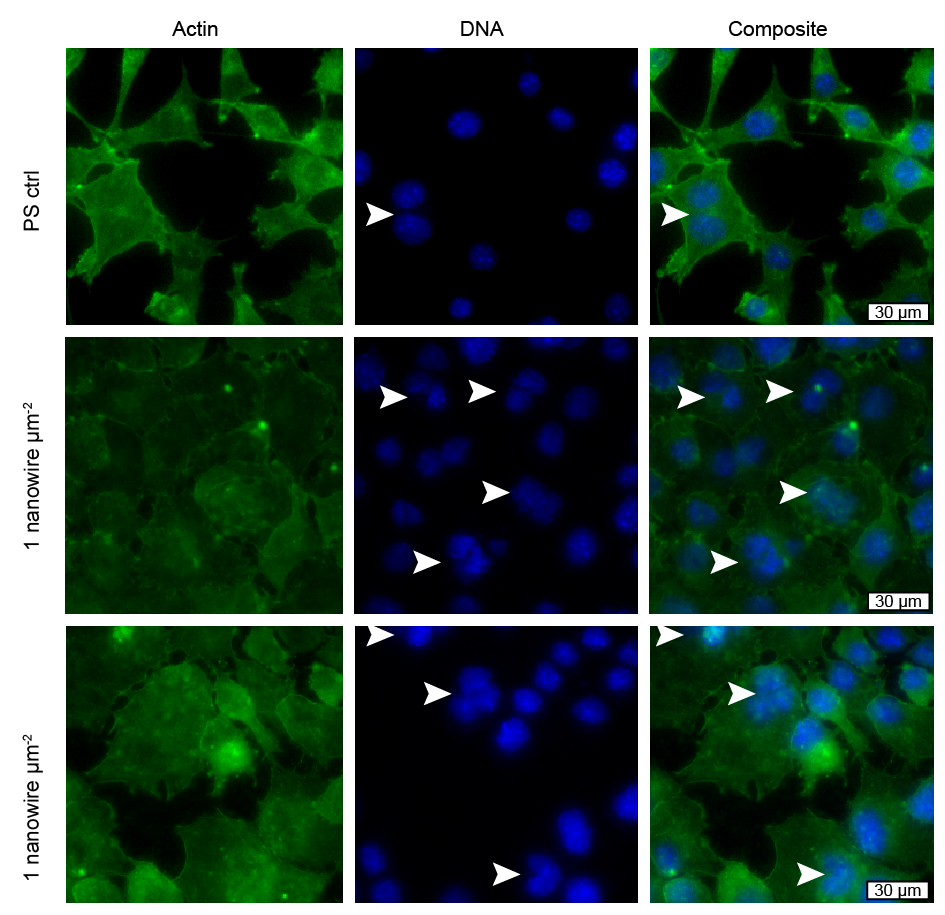


**Figure S3** Examples of cells with multiple nuclei (arrowheads) for. Samples fixed after 96 h culture. Actin is labelled with FITC-conjugated phalloidin (green) and DNA is labelled with Hoechst 33342 (blue).


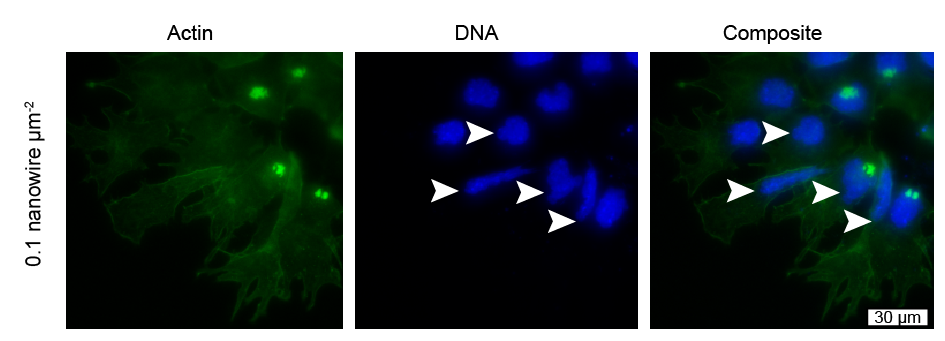


**Figure S4** Examples of jagged nuclei (arrowheads) for. Samples fixed after 96 h culture. Actin is labelled with FITC-conjugated phalloidin (green) and DNA is labelled with Hoechst 33342 (blue).


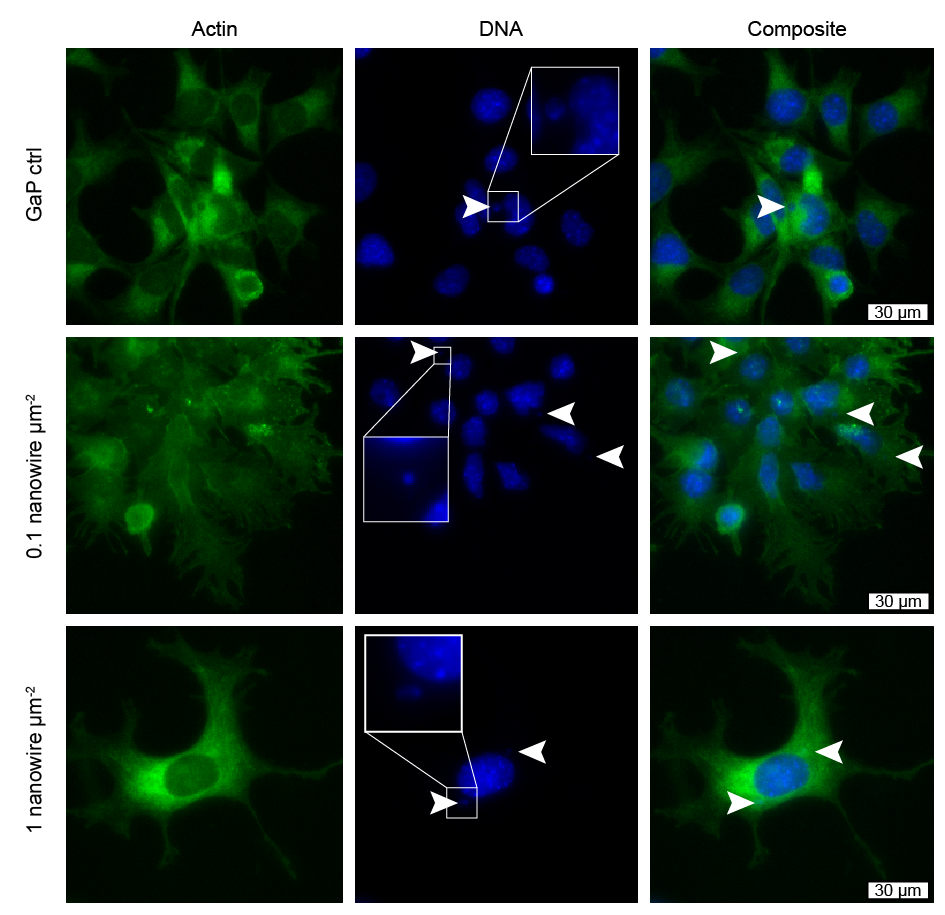


**Figure S5** Examples of micronucleic cells (arrowheads) for samples fixed after 96 h culture. Actin is labelled with FITC-conjugated phalloidin (green) and DNA is labelled with Hoechst 33342 (blue).
